# Latent profile analysis of health-related quality of life in patients with bladder cancer and abdominal wall stomas: a multicenter cross-sectional study

**DOI:** 10.1186/s12894-026-02253-8

**Published:** 2026-07-13

**Authors:** Jiahui Ren, Jingya Lu, Zhufeng Xiong, Yonghui Zhang, Hui Hong, Meiyun Yang

**Affiliations:** 1 School of Nursing, Jiangxi Medical College, Nanchang University, Nanchang, Jiangxi China; 2 Department of Nursing, The 1st Affiliated Hospital of Nanchang University, Nanchang, Jiangxi China; 3 Department of Thoracic Surgery, Henan Provincial Intelligent Nursing and Transformation Engineering Research Center, Henan Provincial Key Medicine Laboratory of Nursing, Henan Provincial People’s Hospital, Zhengzhou University People’s Hospital, Zhengzhou, Henan 450003 China; 4 Emergency, Jiangxi Mental Health Center, Nanchang, Jiangxi China; 5 Intensive Care Unit, Department of Infection, The 1st Affiliated Hospital of Nanchang University, Nanchang, Jiangxi China

**Keywords:** Bladder cancer, Abdominal wall stoma, Health-related quality of life, Patient-reported outcomes, Latent profile analysis, Associated factors

## Abstract

Abdominal wall stomas in bladder cancer patients can severely impact physical, psychological, and social adjustment. However, individual differences in patients’ self-reported outcomes and their influencing factors require further investigation. This multicenter cross-sectional study aimed to identify latent profiles of health-related quality of life among bladder cancer patients with abdominal wall stomas and to examine demographic and clinical factors associated with profile membership.We evaluated 598 patients who underwent radical cystectomy with an abdominal wall stoma between September 2020 and September 2023 in the urology departments of five tertiary hospitals in Beijing, Henan and Jiangxi, China. Data were collected via a self-designed general profile questionnaire and the Chinese Patient Self-Reported Outcome Scale for Bladder Cancer with Abdominal Wall Stoma (PROS-BCAS). Latent profile analysis identified three patient subgroups: “Balanced high-quality type” (*n* = 339, 56.7%), “Unbalanced type” (*n* = 148, 24.7%), and “Low-level type” (*n* = 111, 18.6%). Significant differences were observed across the physical, psychological, social, and treatment-related domains among the three profiles. Multinomial logistic regression showed that lower monthly household income, out-of-pocket medical payment, limited stoma self-care ability, younger age, and complications were associated with membership in poorer health-status profiles. These findings indicate substantial heterogeneity in patient-reported outcomes among bladder cancer patients with abdominal wall stomas. Clinicians should prioritize vulnerable patients with socioeconomic disadvantage, insufficient stoma self-care ability, and complications, and provide targeted economic, educational, psychological, and social support to improve health-related quality of life.

Bladder cancer (BCa) is a malignant tumor that is widespread worldwide [1]. According to the latest data from GLOBOCAN, the number of bladder cancer patients, which ranked 12th, reached 573,000 worldwide in 2020 (GLOBOCAN 2020) [2]. The incidence of bladder cancer is relatively high in Europe and North America, while in East Asia, particularly in China, the incidence and mortality rates are rising significantly, with bladder cancer currently ranked as the seventh most common cancer in the country [3–5].

Muscle-invasive bladder cancer (MIBC), accounting for approximately 25% of all bladder cancer diagnoses, represents an advanced pathological stage characterized by high risks of recurrence and metastatic progression. The standard treatment for MIBC typically involves radical cystectomy(RC) with urinary diversion [6–7], with three main diversion approaches currently in clinical use: orthotopic bladder substitute(OBS), ileal conduit(IC), and cutaneous ureterostomy(CU) [8]. Notably, both IC and CU procedures require permanent external urine collection devices, which frequently result in substantial lifestyle modifications and can significantly compromise multiple dimensions of patient quality of life [9–11].

Nonluminal urinary diversion procedures, such as IC and CU, have been shown in numerous studies to negatively impact patients’ quality of life more significantly than OBS [12]. Physiologically, the need for a permanent stoma and long-term use of a stoma bag can lead to complications such as odor, skin irritation, and urinary tract infections, which interfere with patients’ daily activities [13]. Psychologically, the presence of a stoma can impair patients’ sense of physical integrity, diminish self-image, and contribute to negative emotions, including low self-esteem, anxiety, and depression [14]. Socially, some patients may reduce their interactions with others due to fears of stoma leakage or odor, leading to interpersonal alienation and a lack of social support [15]. Additionally, the ongoing costs of stoma supplies, regular replacement of ureteral stents, and treatment of related complications can place a significant financial burden on patients and their families [16].

To comprehensively assess the health status of patients with an abdominal wall stoma for bladder cancer, researchers worldwide have carried out a series of studies and have made some progress. One study using the EORTC QLQ-C30 scale found that the overall quality of life scores for patients with bladder cancer stomas were significantly lower than those of the general population, particularly in the domains of social and emotional functioning [17]. Another study explored the impact of nursing interventions based on a health belief model, using the Stoma Quality of Life Scale and the Stoma Adjustment Inventory. While the study reported significant differences in adherence rates and complications between the experimental and control groups, no significant difference was observed in quality of life scores [18]. However, existing research on the quality of life of bladder cancer patients with abdominal wall stomas has notable limitations, including small sample sizes and a lack of exploration into individual differences [19].

In China, research focusing on patients with abdominal wall stomas for bladder cancer remains limited. Using a cross-sectional design, Zhang Y et al. [20] explored the relationship between psychosocial adjustment and quality of life in patients with stoma. They validated the Chinese version of the Ostomy Adjustment Inventory and the Stoma-Quality of Life-Chinese Scale and assessed psychosocial adjustment using the Social Support Revalued Scale. The study surveyed 1,053 Chinese patients with intestinal stoma via electronic questionnaires, revealing that the average level of psychosocial adjustment was moderate and that their quality of life was at a medium level. In a related study, Zou Y et al. [21] investigated the impact of abdominal wall stoma on quality of life in bladder cancer patients, highlighting that stoma-related complications and psychological distress significantly affected patients’ physical and social functioning. However, both studies predominantly treated cohorts as homogeneous, neglecting demographic and clinical heterogeneity that may drive divergent quality of life trajectories.

To address these gaps, analytical approaches that account for interindividual heterogeneity are needed. Latent profile analysis (LPA) is a person-centered statistical method that identifies unobserved subgroups based on response patterns across multiple observed indicators. Compared with traditional variable-centered analyses, LPA can reveal clinically meaningful patient profiles that may be obscured when only total scores or average domain scores are examined. In bladder cancer patients with abdominal wall stomas, LPA may help distinguish patients with different patterns of physical, psychological, social, and treatment-related adjustment. Therefore, this study aimed to identify latent profiles of health-related quality of life in this population and to examine demographic and clinical factors associated with profile membership. The findings may provide evidence for developing targeted nursing interventions and optimizing healthcare resource allocation.

## Subjects and methods

### Study subjects

In this study, we employed a cross-sectional survey design to recruit patients who underwent radical cystectomy with an abdominal wall stoma for bladder cancer. We consecutively recruited participants from the urology departments of five tertiary-level hospitals in Beijing, China; Zhengzhou and Xinxiang, Henan Province; and Nanchang, Jiangxi Province, between September 2020 and September 2023. The inclusion criteria were as follows: (1) Pathologically confirmed diagnosis of bladder cancer; (2) Age ≥ 18 years; (3) Undergone radical cystectomy with an abdominal wall stoma; (4) Ability to comprehend and communicate effectively in the language used for the study (Mandarin Chinese). The exclusion criteria were as follows: (1) Presence of other malignant tumors or severe comorbid medical conditions that could significantly impact quality of life or psychosocial adjustment; (2) Cognitive or communication impairments that would hinder participation in the survey; (3) Unwillingness or inability to complete the survey. The study was approved by the Ethics Committee of the hospital, and the subjects signed an informed consent form that followed the ethical guidelines of the Declaration of Helsinki. To determine a reasonable sample size, the following parameters were considered: type of study (cross-sectional survey), statistical parameters (α = 0.05, 1-β = 0.8, *P* = 0.5, d = 0.1*P*), the minimum sample size was calculated using the formula for proportion estimation: n = [*Z*(α/2)²*P*(1 - *P*)] / d²= [(1.96)²0.5(1–0.5)] / (0.05)²≈ 385. Considering a 20% failure rate and the practical requirements of a multicenter study, a total of 610 questionnaires were distributed. Among these, 598 valid questionnaires were recovered, yielding an effective response rate of 98.00%.

### Research methods

We utilized two instruments: a general information questionnaire and the Patient-Reported Outcome Scale for Bladder Cancer with Abdominal Stoma (PROS-BCAS). Our research team designed the general information questionnaire, which comprised two sections: (1) demographic characteristics (age, education level, marital status, per capita monthly family income, and place of residence) and (2) clinical characteristics (medical payment method, status of stoma care, and occurrence of complications). The questionnaire was designed by members of the research team with reference to domestic and international literature and in accordance with the national conditions of China, after several rounds of expert review and revision, and a presurvey was conducted before the formal survey to further optimize the content and language expression of the questionnaire on the basis of the feedback.

The PROS-BCAS scale, developed by Lu J et al. [22], was specifically designed to assess the health status of bladder cancer patients with postoperative abdominal wall stomas. The scale was developed in strict compliance with the COSMIN reporting standards and psychometric principles, with constructs and items determined through literature reviews, qualitative interviews, and expert consultations. The scale was validated across diverse subpopulations, including rural and urban patients, to ensure its applicability and reliability in different settings. Multiple presurveys and validation studies confirmed the scale’s reliability and validity. The scale contains 44 entries organized into four domains (physical, psychological, social, and therapeutic) with two dimensions under each domain. It uses a 5-point Likert scale, with a total score of 176; higher scores indicate better quality of life. The scale demonstrated excellent reliability, with a Cronbach’sα coefficient of 0.954, Spearman–Brown split reliability of 0.977, and test-retest reliability of 0.924. The PROS-BCAS scale is comprised of four domains and eight dimensions, each of which contains a distinct number of entries. As a result, the total scores for each dimension and domain vary. To ensure comparability of scores, standardization is necessary, particularly because the scale includes reverse-scored entries. The scoring procedure is as follows: Entry Scores: For positively scored entries, the original score is used. For reverse-scored entries, the score is calculated as: (highest possible score + 1) - original score. Dimension Scores: The scores of all entries within the same dimension are summed and divided by the number of entries in that dimension to obtain the dimension score.

To address response bias, anonymous questionnaires were administered, trained interviewers clarified questions, and standardized instructions minimized variability. For missing data, the following procedures were applied: Domain-level missing items: Mean imputation was used if < 20% of items were missing within a domain. Variable-level missing data: Variables with < 5% missing values were imputed using the mode (categorical variables) or median (continuous variables). Variables with ≥ 5% missing values were handled via multiple imputation by chained equations (MICE), generating 10 imputed datasets. According to the relevant studies [23–24], when missing values exceed 20%, the data may not be representative of the original distribution. Therefore, questionnaires with more than 20% missing items in any domain were excluded from the study.

### Data collection

One-on-one questionnaires were administered to patients by uniformly trained investigators. For elderly patients with low literacy levels or poor eyesight, face-to-face interviews were used to collect data. If the patients had difficulty understanding the questions during the survey, the investigators provided neutral explanations and filled in the questionnaires according to the patients’ answers. At the end of the survey, the integrity of the questionnaire was checked on the spot, and those who had not filled in the questionnaire or filled in the questionnaire incorrectly were asked to supplement it in time.

### Statistical analysis

The data were entered into EpiData 3.1 software and proofread independently by two researchers. Statistical analysis was performed using SPSS 27.0 and Mplus 8.3. Categorical variables are presented as frequencies and percentages, with group comparisons assessed by chi-square tests. Normally distributed continuous data are expressed as mean ± standard deviation and compared using t-tests or analysis of variance (ANOVA), with post hoc multiple comparisons conducted when appropriate. Non-normally distributed continuous variables are reported as median and interquartile range (IQR) and analyzed using nonparametric tests. Statistical significance was defined as a two-sided *P* < 0.05. Latent profile analysis (LPA) was implemented in Mplus 8.3 using the eight dimensions of the PROS-BCAS as observed indicators. The optimal number of latent profiles was determined according to the Akaike Information Criterion (AIC), Bayesian Information Criterion (BIC), entropy, Lo-Mendell-Rubin adjusted likelihood ratio test (LMR-LRT), Bootstrap Likelihood Ratio Test (BLRT), class size, and clinical interpretability. Profiles comprising less than 5% of the sample were considered insufficiently stable for clinical interpretation. After the optimal latent profile model was determined, multinomial logistic regression was performed to examine demographic and clinical factors associated with latent profile membership, with the Balanced high-quality type used as the reference group. Odds ratios (ORs) and 95% confidence intervals (CIs) were reported.

### Latent profile analysis

Latent profile analysis (LPA) is a multivariate statistical approach used to identify potential subgroups within a study population based on their characteristic patterns [25]. In this study, we constructed an LPA model to examine the heterogeneous characteristics of bladder cancer patients with abdominal wall stomas. The model incorporated patients’ scores across 8 dimensions spanning four domains (physical, psychological, social, and therapeutic) of the self-report scale as observed variables, with latent subgroup membership serving as the categorical outcome. We iteratively tested models starting with one class and incrementally increased the number until convergence failed or fit indices became unavailable. Model selection criteria included: (1) AIC, BIC, adjusted BIC [aBIC], entropy; (2) likelihood ratio tests (Vuong-Lo-Mendell-Rubin [VLMR] and bootstrap likelihood ratio test [BLRT]); (3) interpretability of classes; and (4) practical relevance, requiring each class to comprise *at least 5% of the total sample* to ensure meaningful subgroup representation. Lower AIC, BIC, and aBIC values, along with entropy values closer to 1, indicated superior model fit. A statistically significant VLMR or BLRT (*P* < 0.05) supported the retention of the *k*-class model over the (*k*-1)-class model.

### Ethics approval and consent to participate

This study received ethical approval from the Institutional Review Board of the First Affiliated Hospital of Nanchang University (Approval No. IIT[2023] Linlun Review No. 218) and was conducted in compliance with the ethical principles outlined in the Declaration of Helsinki and its subsequent amendments. Clinical trial number: not applicable. The research design and implementation of scale assessments adhered to the Strengthening the Reporting of Observational Studies in Epidemiology (STROBE) guidelines. Written informed consent was obtained from all participants prior to their inclusion in the study.

## Results

### General information of the patients

The demographic and clinical characteristics of the 598 participants are summarized in Table 1. Over half of the cohort (50.17%, *n* = 300) were aged > 60 years, with 41.97% (*n* = 251) aged 45–60 years. Economically, 40.80% (*n* = 244) relied on out-of-pocket medical payments, and 27.09% (*n* = 162) had a monthly household income per capita < 2000 CNY, indicating financial vulnerability. Clinically, 40.97% (*n* = 245) experienced complications, and 26.09% (*n* = 156) required complete caregiver assistance for stoma management. These findings underscore the need for targeted interventions addressing socioeconomic disparities and clinical complexity in this population.

**Table 1:** General information questionnaire (*n*,%)

Variables	Categories	Sample size/composition ratio
Age (years)	<45	47(7.86%)
	45 ~ 60	251(41.97%)
	>60	300(50.17%)
Educational level	Junior high school and below	208(34.78%)
	High School or College	205(34.28%)
	Bachelor degree or above	185(30.94%)
Marital status	Married	507(84.78%)
	Unmarried	39(6.52%)
	Other (divorced, widowed, etc.)	52(8.70%)
Per capita monthly household income (CNY)	<2000	162(27.09%)
	2000 ~ 4500	198(33.11%)
	>4500	238(39.80%)
Place of residence	Rural	286(47.83%)
	Urban	312(52.17%)
Medical payment method	Out-of-pocket	244(40.80%)
	Medical insurance	354(59.20%)
Status of stoma care	Completely self-care	125(20.90%)
	Partially self-care	317(53.01%)
	Completely self-careless	156(26.09%)
complications	Yes	245(40.97%)
	No	353(59.03%)

### Results of latent profile analysis

The data on the general characteristics and PROS-BCAS scale scores of 598 patients were imported into Mplus 8.3, and a total of five latent class models were established. The fit indices for these models are presented in Table 2. Table 2 shows that with an increase in the number of potential categories, the fitting indicators, such as the AIC, BIC, and aBIC, show an overall decreasing trend, indicating that the model fit gradually improves. However, the decrease becomes flat at 3 categories and above. In addition, the entropy value of the model with 3 categories is the highest, indicating the best certainty of classification. Combined with the BLRT test results, the category 3 model was superior to the category 2 model, whereas the category 4 and above models did not significantly outperform the category 3 model. Considering the balance between statistical fit and clinical interpretability—where overly complex models may lead to overfitting and reduce practical utility—we ultimately selected the 3-category latent profile model. This choice ensures that the identified subgroups are both statistically distinct and interpretable, facilitating targeted clinical interventions.

**Table 2:** Fit metrics for potential profile models for categories 1 to 5

Class	AIC	BIC	Entropy	*P*_LMR_	*P*_BLRT_	classification probability
1	28781.202	28851.499	1.000	-	-	1.000
2	27533.938	27643.777	0.892	0.000	0.000	0.428, 0.572
3	27016.863	27166.245	0.932	0.000	0.000	0.247, 0.186, 0.567
4	26778.333	26967.257	0.888	0.003	0.000	0.294, 0.179, 0.238, 0.289
5	26634.230	26862.697	0.908	0.147	0.000	0.178, 0.239, 0.234, 0.296, 0.053

*AIC* Akaike Information Criterion, *BIC* Bayesian Information Criterion, *Entropy* Measure of classification certainty (values closer to 1 indicate better classification)

P_LMR_ = *P*-value for the Lo-Mendell-Rubin adjusted likelihood ratio test (comparing k vs. k-1 classes)

P_BLRT_ = *P*-value for the Bootstrap Likelihood Ratio Test (comparing k vs. k-1 classes)

**Fig. 1:**
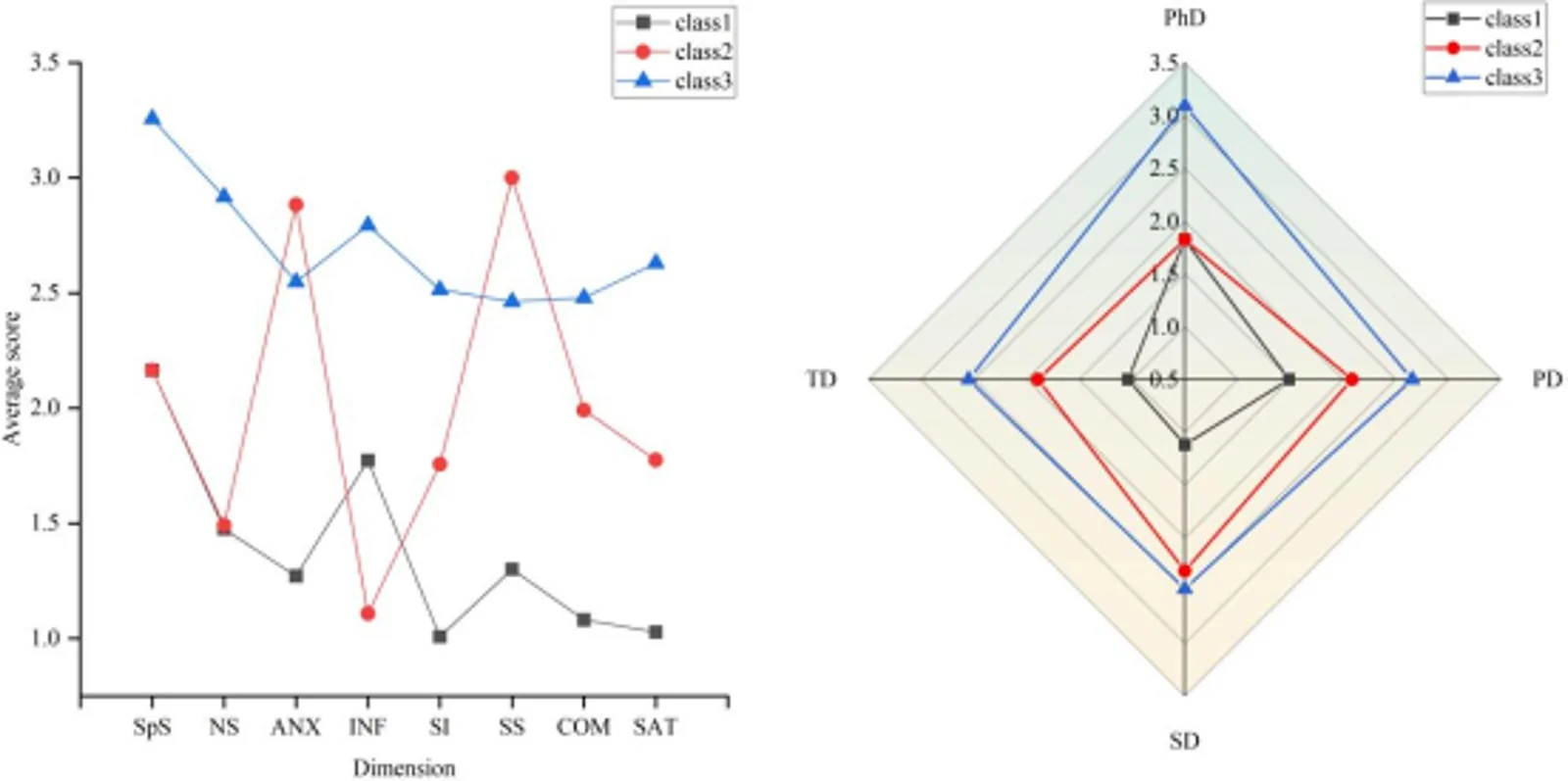
Differences in category scores for bladder cancer patients. Left: potential category score differences across the 8 dimensions; Right: potential category score differences across the 4 domains. Note: PhD = Physiological Domain; PD = Psychological Domain; SD = Social Domain; TD = Treatment Domain; SpS = Specific Symptoms; ANX = Anxiety; INF = Inferiority; SI = Stoma Indication; SS = Social Support; COM = Compliance; SAT = Satisfaction

Figure 1 illustrates the scores of the three patient categories across the eight dimensions and four domains of the PROS-BCAS scale. As shown in the figure, significant differences were observed in the scores across dimensions and domains among the three categories. The left panel reveals that Category 3 patients (56.7%) had the highest scores, indicating that these patients demonstrated strong coping abilities in the physical, psychological, social adaptation, and therapeutic domains. Therefore, this category was labeled as “Balanced high-quality type.” In contrast, Category 1 patients (18.6%) had the lowest scores, suggesting that they faced greater challenges in adapting to and resolving the multifaceted issues caused by their disease. This category was labeled as “Low-level type.” Category 2 patients (24.7%) exhibited relatively imbalanced scores across dimensions and were labeled as “Unbalanced type.” The right panel further highlights the differences in scores across the four domains, confirming significant heterogeneity in the health status of patients in terms of physical, psychological, social adaptation, and therapeutic aspects.

### The scores of the 3 potential categories in different areas

Table 3 shows the differences in the scores of the three potential categories of patients in the four domains of the PROS-BCAS scale and the total score. The results of the ANOVA revealed that the domain scores and total scores were significantly different among the three categories (*P* < 0.05). In the physical domain, the Class 1 and Class 2 scores were not significantly different, but both were significantly lower than those of Class 3. In the psychological, social and therapeutic domains, the scores of the three categories showed a gradient distribution pattern of Class 3 > Class 2 > Class 1 (*P* < 0.05). The comparative results of the total scores were consistent with those of the psychological, social and therapeutic domains, which were also characterized by Class 3 > Class 2 > Class 1 (*P* < 0.05). These findings suggest that there are significant differences among the three potential categories of patients in terms of physical, psychological, and social adaptation and disease management, suggesting that the health status and needs of the bladder cancer stoma population are characterized by significant heterogeneity.

**Table 3:** Differences in the scores of patients in the 3 categories in the 4 domains

Domain	Class1(*n* = 148)	Class2(*n* = 111)	Class3(*n* = 339)	Total
Physiological Domain	21.885 ± 3.840^3^	21.884 ± 5.492^3^	37.080 ± 5.918^1 2^	30.462 ± 9.264
Psychological Domain	18.770 ± 3.561^2 3^	13.393 ± 3.423^1 3^	23.908 ± 4.404^1 2^	20.691 ± 5.760
Social Domain	20.811 ± 4.400^2 3^	10.071 ± 3.440^1 3^	22.367 ± 5.690^1 2^	19.707 ± 6.836
Treatment Domain	26.520 ± 8.066^2 3^	14.536 ± 6.100^1 3^	35.704 ± 7.981^1 2^	29.473 ± 11.176
Total	87.986 ± 12.414^2 3^	59.884 ± 11.039^1 3^	119.059 ± 14.620^1 2^	100.333 ± 26.920

^1^: statistically significant difference from category 1

^2^: statistically significant difference from category 2

^3^: statistically significant difference from category 3

### Analysis of the influencing factors of the three categories of bladder cancer in patients with an abdominal wall stoma

Using the three potential categories as dependent variables (reference: Category 3, “balanced high-quality type”), a chi-square test was performed to assess differences in general characteristics across the classes. Variables with statistically significant differences (*P* < 0.05) were included in a multivariate logistic regression model. Variables were coded as follows: Age: <45 years = 1, 45 ~ 60 years = 2, >60 years = 3 (reference: >60 years). Per apita monthly household income: <2000 CNY = 1, 2000 ~ 4500 CNY = 2, > 4500 CNY = 3 (reference: >4500 CNY). Medical payment method: Out-of-pocket = 1, Medical insurance = 2 (reference: Medical insurance). Status of stoma care: Completely self-care = 1, Partially self-care = 2, Completely self-careless = 3 (reference: completely self-careless). Complications: Yes = 1, No = 2 (reference: No complications), the results are shown in Table 4.

**Table 4:** Multiple logistic regression analysis of factors influencing the potential categories

Variables		Class1				Class2			
		OR	Waldχ^2^	*P*	95%CI	OR	Waldχ^2^	*P*	95%CI
Age(Years)	<45	0.251	28.740	0.003	0.100 ~ 0.622	0.998	0.000	0.988	0.800 ~ 1.245
	45 ~ 60	0.114	9.970	0.002	0.029 ~ 0.438	1.001	0.000	0.990	0.850 ~ 1.179
Average monthly Income per Household (CNY)	<2000	10.704	10.42	0.001	2.538 ~ 45.137	3.253	0.000	0.052	1.200 ~ 8.812
	2000 ~ 4500	8.095	14.79	<0.001	2.789 ~ 23.500	10.119	23.560	<0.001	3.975 ~ 25.762
Healthcare cost Payment method	Out-of-pocket	5.803	12.17	0.001	2.154 ~ 15.656	4.206	0.000	0.980	1.652 ~ 10.701
Status of stoma care	Completely self-care	8.905	17.550	<0.001	3.172 ~ 24.391	6.428	0.000	0.590	2.510 ~ 18.500
	Partially self-care	2.801	2.410	0.120	0.764 ~ 10.269	0.598	1.410	0.235	0.256 ~ 1.397
Complications	Yes	0.154	10.850	<0.001	0.052 ~ 0.453	1.004	0.000	0.980	0.820 ~ 1.229

**Fig. 2:**
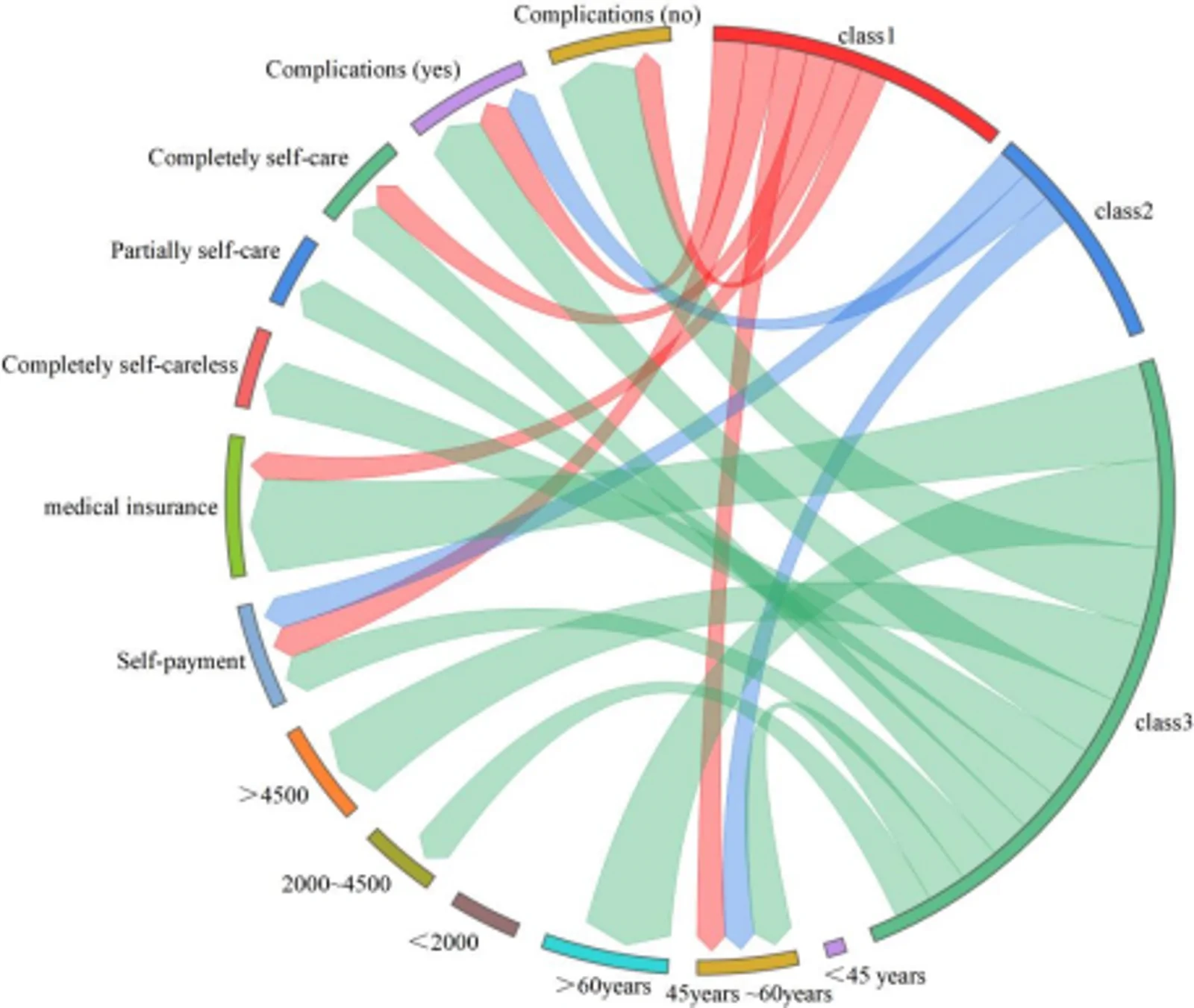
Correlation analysis of the 3 categories of patients’ general information

The findings revealed that, compared to Class 3 (“Balanced high-quality type”), Class 1 (“Low-level type”) patients were more likely to be younger than 60 years, have a monthly household income per capita of less than 4500 CNY, pay out-of-pocket for medical expenses, be completely dependent on others for stoma care, and have complications. Multivariate logistic regression analysis and the correlation analysis in Fig. 2 indicated that Class 3 (“Balanced high-quality type”) patients were more likely to be older than 60 years, have a monthly household income per capita of more than 4500 CNY, use medical insurance for payment, be completely independent in stoma care, and have no complications. For Class 2 (“Unbalanced type”), no statistically significant associations were observed for age (45–60 years: *P* = 0.988), stoma care ability (Partially self-care: *P* = 0.235), or complications (*P* = 0.980). The sole significant predictor for this group was per capita monthly household income (2000–4500 CNY vs. >4500 CNY; *P* < 0.001).

Figure 2 presents a chord diagram illustrating the distribution of general characteristics among patients in the three categories. The diagram provides an intuitive visualization of the differences in general demographic features across the three patient groups. The results displayed in the chord diagram are consistent with the findings from the multivariate logistic regression analysis, suggesting that factors such as age, family economic status, medical payment methods, stoma care conditions, and comorbidities may influence the health status of bladder cancer patients. These factors could contribute to the expression of distinct characteristics in reported health outcomes, leading to the formation of different latent classes.

## Discussion

This multicenter cross-sectional study identified three distinct latent profiles of health-related quality of life among bladder cancer patients with abdominal wall stomas: the Low-level type, the Unbalanced type, and the Balanced high-quality type. These profiles differed significantly across the physical, psychological, social, and treatment-related domains of the PROS-BCAS, indicating substantial heterogeneity in patient-reported health status. This finding suggests that bladder cancer patients with abdominal wall stomas should not be regarded as a homogeneous population. Instead, their multidimensional health-related quality of life should be understood through profile-specific patterns, which may provide a clinically meaningful basis for targeted nursing care.

The three-profile solution provides a nuanced understanding of the health status of this population. Patients in the Balanced high-quality type showed relatively favorable scores across all domains, suggesting better adaptation to postoperative stoma-related changes and stronger coping capacity in daily life. In contrast, patients in the Low-level type had consistently lower scores, reflecting broad difficulties in physical symptoms, psychological adjustment, social participation, and treatment-related adaptation. These patients may represent the most vulnerable subgroup requiring comprehensive and continuous support. The Unbalanced type showed an intermediate and uneven pattern, indicating that some patients may not experience global impairment but may have specific deficits in certain domains. Therefore, interventions should not only focus on patients with globally poor quality of life but should also identify domain-specific weaknesses among patients with unbalanced profiles.

Low per capita monthly household income and out-of-pocket medical expenses may significantly impact the prognosis of patients in multiple ways. Recent studies highlight the complex interplay between socioeconomic factors and cancer health outcomes. For instance, Joyce DD et al. [26] demonstrated that financial constraints often lead to delays in seeking medical care and adhering to treatment regimens, resulting in missed early intervention opportunities and worsened disease progression. This aligns with our findings that patients with lower household incomes and self-payment for medical costs reported higher levels of psychological distress and impaired social functioning. Furthermore, Van Beek FE et al. [27] found that financial burdens strongly correlate with increased anxiety and depression among cancer patients, adversely affecting their quality of life and treatment outcomes. This is consistent with our observation that economic hardship exacerbates psychological distress in bladder cancer patients with abdominal stomas. Additionally, Rosskamp M et al. [28] reported that socioeconomic disadvantages, including low income, link to unhealthy lifestyle behaviors like smoking and excessive alcohol consumption, further compromising patient prognosis. These findings underscore the multifaceted impact of economic status on patient outcomes through mechanisms such as healthcare access, psychological well-being, and lifestyle choices. In conclusion, economic status appears to play a role in influencing the prognosis of bladder cancer patients through various dimensions, including medical behaviors, psychological health, and lifestyle factors. Addressing these socioeconomic determinants is crucial for improving patient outcomes and requires targeted interventions.

Our findings regarding the relationship between age and prognosis are consistent with those of Zhang Y et al. [29], who reported that middle-aged patients aged 45–60 years exhibited a significantly lower quality of life compared to other age groups. Sritan S et al. [30] further supported this observation, noting that middle-aged patients with stomas experienced less improvement in quality of life than older patients, emphasizing the need for tailored nursing care to address their specific needs. Additionally, Eklöv K et al. [31] highlighted that middle-aged patients often face a reduction in their social circles and decreased interactions with friends and colleagues, leading to diminished social support. This sense of isolation, coupled with increased reliance on support systems, may exacerbate feelings of helplessness and anxiety, potentially hindering their recovery.In conclusion, middle-aged patients aged 45–60 years may encounter unique challenges in their physical, psychological, and social roles, underscoring the importance of providing targeted care and guidance to meet their specific needs.

Stoma care had a significant effect on patient classification, with “Low-level type” patients generally showing complete inability to care for themselves (*P* < 0.05). As a highly specialized treatment operation, stoma care requires patients to have appropriate knowledge of the disease and nursing skills [32]. However, patients with an abdominal wall stoma for bladder cancer are mostly elderly patients, often characterized by cognitive and memory loss [33]. Therefore, patients who are completely unable to care for themselves are unable to perform daily stoma cleaning and care independently, which may lead to skin problems due to improper use of stoma devices. In addition, dietary restrictions and psychological problems may lead to malnutrition and decreased immunity, which in turn may affect the recovery process and prolong the prognosis. Patients who are completely unable to take care of themselves need to rely on the help of others, and patients may face psychological burden and stress, which in turn affects overall recovery [34]. Therefore, providing a professional nursing team, effective rehabilitation measures and psychological support is crucial. These measures not only reduce the risk of infection and improve skin condition but also provide nutritional support, reduce psychological stress, and promote patients’ social participation, thus significantly improving their prognosis.

From a clinical perspective, the present findings support a profile-based approach to nursing assessment and intervention. Medical staff can use the identified associated factors, such as low income, out-of-pocket medical payment, limited stoma self-care ability, complications, and younger age, as practical indicators for identifying patients who may require additional support. For patients in the Low-level type, comprehensive interventions including economic support, stoma care training, complication management, psychological counseling, and social support should be prioritized. For patients in the Unbalanced type, interventions should focus on the specific domains in which patients show poorer adjustment, such as psychological adaptation, social reintegration, or treatment-related management. Patients in the Balanced high-quality type may still require routine follow-up to maintain their current level of adaptation and prevent deterioration. This profile-based approach may help shift postoperative care from generalized management to more individualized and targeted nursing support.

From a research perspective, this study extends previous quality-of-life research in bladder cancer patients with abdominal wall stomas by shifting from a variable-centered approach to a person-centered approach. Traditional analyses often examine average associations between individual factors and quality-of-life scores, which may obscure meaningful heterogeneity within the patient population. In contrast, LPA identifies naturally occurring subgroups based on response patterns across multiple domains. This approach provides a more detailed understanding of how physical, psychological, social, and treatment-related dimensions cluster within individuals. Such evidence may support the development of more precise assessment strategies and subgroup-specific nursing interventions in future research.

### Limitations of the study

While this study provides valuable insights into bladder cancer patients with abdominal wall stomas, several limitations warrant careful consideration. The cross-sectional design inherently precludes causal inferences, and reliance on self-reported data may introduce recall bias. Although prospective cohort studies tracking temporal changes could better establish temporal relationships between associated factors and health-related quality of life profiles, observational longitudinal designs may still be affected by residual confounding and unmeasured variables. Therefore, future research should incorporate longitudinal designs to examine whether patients transition between different latent profiles over time and to further clarify the factors associated with improvement or deterioration in health-related quality of life. Regarding generalizability, although our multi-regional study included patients from five hospitals in Beijing, Henan, and Jiangxi with certain socioeconomic diversity, the sample still lacks national representativeness; expansion to western regions, rural areas, and different levels of healthcare settings, such as community hospitals and primary care institutions, would strengthen the external validity of the findings. In addition, resource constraints limited the collection of long-term follow-up data and objective physiological indicators. Future studies could integrate electronic health records, routine follow-up systems, and mobile health platforms to enable continuous post-discharge monitoring and more comprehensive outcome assessment. Finally, although the three-profile solution showed good statistical fit and clinical interpretability, the stability and applicability of these latent profiles should be further validated in independent cohorts, and future intervention studies are needed to explore whether profile-specific nursing strategies can improve health-related quality of life among bladder cancer patients with abdominal wall stomas.

## Conclusion

This study identified three distinct latent profiles of health-related quality of life among bladder cancer patients with abdominal wall stomas: the Low-level type, the Unbalanced type, and the Balanced high-quality type. Lower monthly household income, out-of-pocket medical payment, limited stoma self-care ability, younger age, and complications were associated with poorer profile membership. These findings indicate substantial heterogeneity in patient-reported outcomes and emphasize the need for stratified care strategies. Clinicians should prioritize vulnerable subgroups by providing economic support, optimizing medical insurance and reimbursement guidance, strengthening individualized stoma care education, preventing and managing complications, and offering targeted psychological and social support. Future longitudinal studies should validate the stability of these latent profiles in diverse regions and healthcare settings, examine changes in health-related quality of life over time, and develop profile-specific nursing interventions to further improve care for bladder cancer patients with abdominal wall stomas.

## Data Availability

The datasets used and/or analyzed during the current study are available from the corresponding author upon reasonable request.
